# Mobile Sensing in the COVID-19 Era: A Review

**DOI:** 10.34133/2022/9830476

**Published:** 2022-08-07

**Authors:** Zhiyuan Wang, Haoyi Xiong, Mingyue Tang, Mehdi Boukhechba, Tabor E. Flickinger, Laura E. Barnes

**Affiliations:** ^1^School of Engineering and Applied Science, University of Virginia, Charlottesville, USA; ^2^Big Data Lab, Baidu Research, Baidu Inc., BeijingChina; ^3^Department of Medicine, University of Virginia, Charlottesville, Virginia, USA

## Abstract

*Background*. During the COVID-19 pandemic, mobile sensing and data analytics techniques have demonstrated their capabilities in monitoring the trajectories of the pandemic, by collecting behavioral, physiological, and mobility data on individual, neighborhood, city, and national scales. Notably, mobile sensing has become a promising way to detect individuals’ infectious status, track the change in long-term health, trace the epidemics in communities, and monitor the evolution of viruses and subspecies.*Methods*. We followed the PRISMA practice and reviewed 60 eligible papers on mobile sensing for monitoring COVID-19. We proposed a taxonomy system to summarize literature by the *time duration* and *population scale* under mobile sensing studies.*Results*. We found that existing literature can be naturally grouped in *four clusters*, including *remote detection*, *long-term tracking*, *contact tracing*, and *epidemiological study*. We summarized each group and analyzed representative works with regard to the system design, health outcomes, and limitations on techniques and societal factors. We further discussed the implications and future directions of mobile sensing in communicable diseases from the perspectives of technology and applications.*Conclusion*. Mobile sensing techniques are effective, efficient, and flexible to surveil COVID-19 in scales of time and populations. In the post-COVID era, technical and societal issues in mobile sensing are expected to be addressed to improve healthcare and social outcomes.

## 1. Introduction

Since 2019, the coronavirus disease, namely, COVID-19 or SARS-CoV-2, has already rampaged across the world for more than two years and caused the death of over 5 million people (https://covid19.who.int/). With the wide adoption of customer electronics and personal mobile devices over large populations in this century, COVID-19 has become the first-ever global pandemic surveilled digitally. Among these digital surveillance methods, mobile sensing leveraging embedded sensors in mobile devices (e.g., smartphones [1] and wearables [2]) becomes a pervasive way to collect human physiological, behavioral, and environmental data, and trace the human interactions in multiple spatial levels [3]. There are several technical reviews for mobile-enabled technology and data science for healthcare [3– 6], especially for the COVID-19 control [1, 7– 12]; some also focus on specific COVID-19-caused issues (e.g., mental health support) [13, 14]. However, while these articles cover the use of mobile devices to respond to COVID-19, there is still a lack of a review paper investigating the study design, expected health outcomes, and existing limitations of such mobile-based human-subject studies to guide future practice.

In this paper, we conduct a literature review that covers scholarly works leveraging mobile sensing systems to monitor the individual or population health status related to COVID-19. Specifically, we conduct a comprehensive search to retrieve related publications using a set of well-designed keywords from multiple databases and select eligible papers with refining strategies to focus on the results obtained from human-subject studies or clinical trials. Then, we summarize the health outcomes achieved by these publications, where we are interested in how such studies are designed, particularly how many human subjects (*population scale*) and how long (*time duration*) these mobile sensing studies have covered.

We map selected works into a two-dimensional taxonomy system based on the *time duration* and *population scale*, where these works are naturally grouped into *four* clusters, standing for (1) *remote detection* (N=15) that identifies individual’s infection status just in time, (2) *long-term tracking* (N=7) that continuously monitors individual’s health status from infection, incubation, symptom onset to recovery, or exacerbation, (3) *contact tracing* (N=11) which helps understand how COVID-19 spreads among neighborhoods via in-person interactions within days and weeks, and (4) *epidemiological study* (N=27) that reveals the dynamics of virus variants and spread globally through worldwide mobile sensing data collection. We further analyze the technical design, health outcomes, and limitations of each cluster of mobile sensing techniques and discuss the intracluster variations among these techniques due to the use of different sensory data. Finally, we conclude this review and discuss the implications and research directions of the technology (i.e., mobile sensing data and scalable systems) and applications (i.e., for clinicians, healthcare, and policy making).

## 2. Methods

This study was designed and implemented through following the methods of the existing review and report [15, 16], where we follow the rapid review method proposed in [15] to accelerate the review process and report our findings under the preferred reporting items for systematic reviews and meta-analysis (PRSIMA) framework [16].

### 2.1. Design of the Study

Mobile sensing studies involve human subjects to collect sensory data streams over individuals and time slots; it also has been applied to conduct data analyses and knowledge discovery for potential outcomes. Significantly, the design of the population scale and time duration plays a crucial role in such studies, as improper or unrealistic population and time coverage will lead to unreliable analytics results, heavy resource consumption, and poor health outcomes. In this study, we aim to investigate the designs of such studies from the population scale and time duration of two perspectives; moreover, what are the outcomes and limitations? Particularly, we aim to search eligible literature which answers the questions as follows:

(i) What are the aims and designs of mobile sensing techniques for COVID-19
(ii) What health outcomes could be expected by mobile sensing techniques for COVID-19
(iii) For various techniques, what sensing duration should the measurements be to track the status of a user
(iv) For expected health outcomes, what population scales should the study design cover and what data should be collected
(v) What are the limitations of existing mobile sensing techniques for COVID-19

### 2.2. Search Strategy and Selection Process

To answer the above-highlighted research questions, we first conducted a comprehensive search of English literature from databases (i.e., PubMed, Google Scholar, ScienceDirect, Web of Science, and NCBI) under the following keyword combination: (‘mobile sensing’) ∗AND (‘COVID-19’) from December 2019 to March 2022. Then, we iteratively updated the keywords based on COVID-19 (e.g., SARS-CoV-2 and pandemic) and mobile sensing-related (e.g., mobile device/app/system) keywords. Furthermore, several additional items were also considered in the literature search (e.g., spread and monitoring). The final list of keywords is presented in Table 1. To avoid missing crucial articles that might not be timely indexed by the databases or covered by the keywords, we also retrieved articles from the websites of top-tier conferences (e.g., SIGKDD Conference on Knowledge Discovery and Data Mining, AAAI Conference on Artificial Intelligence, and Annual International Conference on Mobile Computing And Networking) and journals (e.g., Nature, Science, Lancet, and their partner journals). 

**Table 1:** Keywords for literature search after iterative updates.

Mobile sensing related	COVID-19 related	Additional items
Mobile sensing	COVID-19	Spread
Mobile device, mobile app, mobile system	SARS-CoV-2	Transmission
Mobile phone, cell phone, smartphone	Coronavirus	Tracking
Wearable, smartwatch	Pandemic	Tracing
Mobility		Monitoring
Mobile health		

After removing duplicates, we first adopted title/abstract screening to exclude irrelevant papers. Then, we used the eligibility criteria to evaluate the detailed full text and relevance of included papers, and finally, the studies that utilize mobile sensing techniques to collect data over a certain sensing time duration and population scale to address COVID-19-related issues are retained. The details of eligibility criteria include the following: (1) the study must be associated with COVID-19 issues, (2) the study should involve human subjects to evaluate the effectiveness of mobile sensing techniques for COVID-19-related outcomes, (3) the study should report the *time duration* and *population scale* that mobile users should be involved to achieve the target health outcome, and (4) the study should report subject measures on health outcomes achieved by mobile sensing techniques. Two authors (Z. Wang and M. Tang) separately conducted the selection processes and reached the final consensus by fully discussing conflicts or disagreements that occurred during this process. 

### 2.3. Data Collection and Synthesis

For each included study, we clearly distinguished the type(s) of mobile data sensed and its sensing time duration and population scale in the data description part. Then, we mapped all the included studies onto a taxonomy system based on the sensing duration and population scale, to classify and understand the distribution of sensed data in these studies. To be specific, according to the commonly selected sensing duration and population scale selected in these practices, the sensing duration was divided into the scales of just in time (seconds, minutes, and other time slots within a day), days, months, and years; the population scale was mapped into the following levels: individual, neighborhood/city, state/nation, and multination/global levels. Finally, we summarize the identified categories of mobile sensing in the COVID-19 era work and introduce the representative works while highlighting their health outcomes, sensor data types, and time duration and population scale that they covered.

## 3. Results

### 3.1. Study Selection

**Figure 1:**
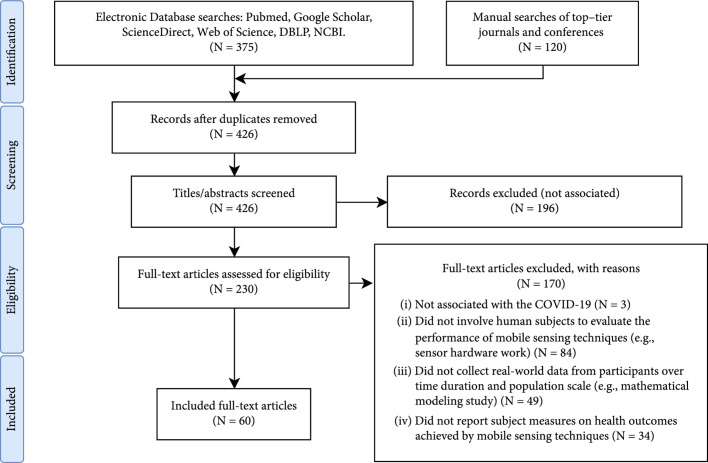
Study selection process under PRISMA framework.

Our search resulted in a total of 495 records, where 375 records were from electronic databases and 120 records were manually searched from top-tier journals and conferences. 426 were further screened after removing 69 duplicates. Then, 196 records were excluded after title and abstract screening. Under proposed eligibility criteria, 177 records were further excluded, where 3 were not associated with COVID-19, 84 did not involve human subjects in the mobile sensing practices (e.g., system work with no human-in-the-loop data collection), 49 did not collect data from participants (e.g., mathematical modeling and simulation study), and 34 did not report subject measures on health outcomes achieved by mobile sensing techniques. Finally, 60 records met the eligibility criteria (see Figure 1). 

### 3.2. Study Characteristics

The 60 eligible studies, as shown in Table 2, deploy mobile sensing techniques to monitor mobile users at a varying *population scale* (22 were at the individual level, 25 were for neighborhoods/cities, 9 were at the state/national level, and 4 were globally) and in multiple levels of *time duration* (12 were just in time, 17 were at the level of days, 30 were months, and 1 was for years), so as to primarily obtain various types of sensory data (9 physiological, 8 audio, 32 GPS records or call detailed records (CDRs), 5 Bluetooth proximity, 5 self-reported survey, and 3 others). 

**Table 2:** Characteristics and distribution of the selected studies.

Characteristics	N	%
Sensing duration		
Just in time	12	20.0
Days	17	28.3
Months	30	50.0
Years	1	1.7
Population scale		
Individual	22	36.7
Neighborhoods/city	25	41.7
States/nation	9	15.0
Multinations/global	4	6.7
Sensor data types		
Physiological	9	15.0
Audio	8	13.3
GPS locations or call detailed records (CDRs)	32	53.3
Bluetooth proximity	5	8.3
Self-reported survey	5	8.3
Others (e.g., WiFi and social media)	3	5.0

### 3.3. Results of Studies Clustered by the Taxonomy System

**Figure 2:**
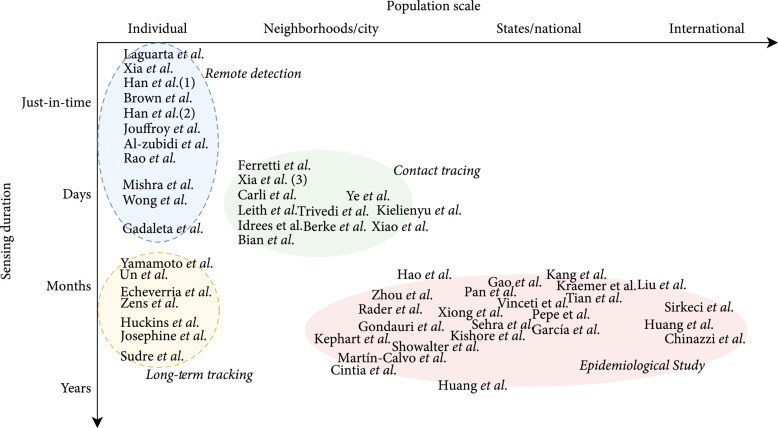
Mapping literature to a two-dimensional taxonomy.

As every study reports the *population scale* and *time duration* that mobile sensing techniques have covered, we map these studies onto a taxonomy system based on the time duration and population scale for mobile sensing. As shown in Figure 2, the selected papers were naturally grouped in *four clusters*. After taking a closer look at every cluster, we summarize the *aims* of mobile sensing techniques in every cluster as follows:

(1) *Remote detection*$\left(N=15,25.0\%\text{of}\text{studies}\right)$ leverages microphones or wearable sensors to collect acoustic signals and physiological data from individuals and identify the just-in-time infection status of COVID-19
(2) *Long-term tracking* (N=7, 11.7% of studies) collects users’ self-reported symptoms and physiological data to continuously monitor changes in the individual’s health status from infection, asymptotic status, symptom onset to recovery, or progression
(3) *Contact tracing* (N=11, 18.3% of studies) collects Bluetooth proximity records or GPS trajectories to track in-person interactions between mobile users and to identify the spread of COVID-19 among neighborhoods
(4) *Epidemiological study* (N=27, 45.0% of studies) performs long-term surveillance on human mobility of large crowds to reveal the dynamics of virus spread

Table 3 summarizes the sensing duration, population scale, human data collected, and achieved health outcomes of the four clusters. In the next step, we perform a synthesis analysis of mobile sensing techniques in the four clusters. 

**Table 3:** The high-level summary of the four types of mobile sensing applications in COVID-19.

Category	Sensing duration	Population scale	Data type	Outcomes
Remote detection (N=15)	Just in time to days	Individual	Respiratory audio data, physiological data	Remotely detecting the symptomatic or presymptomatic signs of infection
Long-term tracking (N=7)	Days to months	Individual	Physiological data, self-reported symptoms	Continuously monitoring the progressions of physiology and symptoms of patients
Contact tracing (N=11)	Days (about 2 weeks)	Neighborhood to community	Bluetooth proximity, GPS locations, CDRs	Automatically identifying the individuals or locations with high exposure risk
Epidemiological study (N=27)	Months to years	Community to international	Mobility data (e.g., GPS trajectories, CDRs, and social media check-ins)	Comprehensively understanding the spread and its mechanism from the perspective of human mobility

### 3.4. Results of Synthesis Analysis

We present our analysis on every cluster of mobile sensing techniques for COVID-19 from the perspectives of system design, health outcomes, and their positive/negative impacts on social factors.

#### 3.4.1. Remote Detection

*(1) Design*. Remote detection (see in Table 4) majorly leverages (1) *audio data*, e.g., respiratory sounds collected from microphones equipped in smartphones (N=8 of 15) [17– 24], and (2) *physiological data*, e.g., blood oxygen sensed by smartwatches (N=6 of 15) [25– 30], to identify whether a user has been infected through machine learning-based approaches. Some of them [20,28,30,31] also leverage/incorporate *self-reported symptom* data to improve the effectiveness of detection. In practice, the predictive models are trained using datasets collected from a large group of both positive and negative cases, where the model learns discriminative features from sensory data and/or self-reported symptom records.

**Table 4:** Study characteristics of remote detection literature.

Reference	Data type	Population scale	Sensing duration	Description
Kumar and Alphonse [17]	Audio	Individual	Just in time	Detecting the symptoms of COVID-19 from crowd-sourced sound data
Laguarta et al. [18]	Audio	Individual	Just in time	Detecting asymptomatic COVID-19 from cough recording data
Xia et al. [19]	Audio	Individual	Just in time	Detecting respiratory and COVID-19 symptoms from crowd-sourced sound data
Han et al. [20]	Audio	Individual	Just in time	Detecting COVID-19 from the combination of self-reported symptoms and sounds
Brown et al. [21]	Audio	Individual	Just in time	Detecting COVID-19 from multiple respiratory sound data
Han et al. [22]	Audio	Individual	Just in time	Exploring the realism and societal bias of detecting COVID-19 from sound data
Ismail et al. [23]	Audio	Individual	Just in time	Evaluating the signatures of COVID-19 from the vibrations of the vocal folds
Orlandic et al. [24]	Audio	Individual	Just in time	Enabling large-scale COVID-19 screening based on cough recordings
Teo [25]	Physiological	Individual	Just in time	Detecting COVID-19 using smartphone built-in pulse oximeters
Jouffroy et al. [26]	Physiological	Individual	Just in time	Detecting COVID-19 from early abnormalities of silent hypoxemia
Al-zubidi et al. [27]	Physiological	Individual	Just in time	Using machine learning approaches to classify influenza and COVID-19
Mishra et al. [28]	Physiological	Individual	Days	Detecting the presymptomatic COVID-19 from instant smartwatch data streams
Wong et al. [29]	Physiological	Individual	Days	Using wearable biosensors to monitor the physical condition for early detection
Gadaleta et al. [30]	Physiological	Individual	Days	Detecting the onset of COVID-19 from wearable sensors and self-reported symptoms
Rao and Vazquez [31]	Self-report	Individual	Just in time	Detecting COVID-19 using mobile-based self-reported web surveys

*(2) Outcomes*. In terms of health outcomes, existing studies evaluate the effectiveness of proposals through binary classification between positive/negative infectious statuses. For example, Gadaleta et al. [30] exhibited an AUC of 0.83 with a predictive model leveraging both *physiological data* and *self-reported symptoms*, where they evaluate their proposals using a dataset collected from 1118 positive and 7032 negative samples. On the other hand, the methods using *acoustic signals* [20,28] could achieve an AUC range from 0.71 to 0.97. Note that the assessment of classification accuracy in some studies might be inaccurate. For example, many asymptomatic cases might not be tested and counted in [28], resulting in an underestimate of positive samples.

*(3) Limitations*. The prerequisite of owning wearable devices and the ability to self-report COVID-19-related symptoms might raise the economic and education bars of technology adoption for remote detection [32], resulting in *biases in population coverage*. Furthermore, all these methods rely on large-scale data collection for training datasets, while unbalanced data collections might cause also *biases in prediction results* under varying demographics, languages, devices, and physiological/respiratory conditions [22,33].

Overall, the quality of data and population coverage of wearable devices depends on the technology literacy and health literacy of the devices’ users. It is important to note that morbidity and mortality of COVID-19 has disproportionately affected communities that are socioeconomically disadvantaged, where lower tech and health literacy may also be more prevalent [34,35]. Therefore, there is a risk that reliance on mobile sensing for remote detection could further worsen inequity and health disparities by under-representing the individuals and communities who are actually most impacted by the pandemic.

#### 3.4.2. Long-Term Tracking

*(1) Design*. Long-term tracking (see also in Table 5) majorly leverages *(1) self-reported symptoms* (N=4 of 7) [36– 39] and (2) *physiological data* (N=3 of 7) [40– 42] to continuously monitor individuals’ health status, capture the rapid deterioration of symptoms, and provide adaptive interventions for health. In addition to detect the infectious status, a rising health concern named long-COVID (or, namely, post-COVID conditions) surges the needs of long-term tracking, as some patients of long-COVID would still suffer from prolonged physical, neurological, and cognitive symptoms in a considerable time (https://www.cdc.gov/coronavirus/2019-ncov/long-term-effects/index.html).

**Table 5:** Study characteristics of long-term tracking literature.

Author	Data type	Population scale	Sensing duration	Description
Yamamoto et al. [36]	Self-report	Individual	Weeks	Demonstrating symptom-tracking app and analyzing the health observations
Echeverria et al. [37]	Self-report	Individual	Months	Developing a mobile app to monitor the physical condition of suspected cases
Zens et al. [38]	Self-report	Individual	Months	Identifying and estimating COVID-19 symptoms using self-reported data
Huckins et al. [39]	Self-report	Individual	Months	Analyzing human physical and mental health via StudentLife mobile application data
Un et al. [40]	Physiological	Individual	Weeks	Analyzing physiological data from wearables to automatically track COVID-19
Vogel et al. [41]	Physiological	Individual	Months	Developing TrackYourHealth to respond to COVID-19 with mHealth monitoring
Josephine et al. [42]	Physiological	Individual	Months	Using electronic wearables to early detect and monitor the symptoms of COVID-19

*(2) Outcomes*. Incorporating self-reported symptoms, several works [38,40,43] have studied ways to identify risk factors of long-COVID and crucial progressions. For instance, Sudre et al. [43] studied 4182 users among whom 558 users’ symptoms lasted more than 4 weeks, 189 lasted more than 8 weeks, and 95 lasted more than 12 weeks. The study also indicated that long-COVID could be characterized by symptoms including fatigue, headache, dyspnea, and anosmia, where obese elders might be more susceptible and should get more prevention. Additionally, a study of 11829 participants completed a questionnaire based on symptoms and underlying conditions identified that the most significant risk factors for exacerbations were diabetes and chronic heart disease [38]. With physiological signals such as oxygen saturation, respiratory rate, heart rate, and skin temperature, some works leverage novel wearable sensors with machine learning models to automatically detect clinical deterioration [40]. After all, it is difficult to evaluate the outcomes of long-term tracking measures, as progression or recovery might be also affected by many other factors in the long term [44].

*(3) Limitations*. A major concern of symptom-based methods is the accuracy of the self-reported symptoms and the effectiveness of using self-reported symptoms to identify disease progression. As individuals’ ability of symptom checking or self-diagnosing may vary [45,46], education again becomes a key factor to ensure the quality of health outcomes here. In addition, one major limitation of self-reported methods is the added burden and the resulting low adherence that can affect data quality and utility.

Moreover, mobile sensing for long-term tracking and contact tracing raise similar concerns as remote detection, regarding dependence on tech literacy and health literacy for accurate data and representative reach into populations. Data collection and prediction favoring communities of higher socioeconomic status pose risk of worsening inequity and health disparities for those who are not included [47,48].

#### 3.4.3. Contact Tracing

*(1) Design*. For COVID-19, contact tracing systems leverage (1) *proximity-based footprints*, e.g., encounters between two Bluetooth devices (N=6 of 11) [49– 54], and (2) *location-based footprints*, i.e., GPS trajectories, call detailed records (CDRs), or WiFi sensors (N=5 of 11) [55– 59], to identify in-person interactions or colocation events between mobile users and track the potential spread of COVID-19 among neighborhoods within the duration of the incubation period, as shown in Table 6.

**Table 6:** Study characteristics of contact tracing literature.

Author	Data type	Population scale	Sensing duration	Description
Dar et al. [49]	Bluetooth	Neighborhood	Weeks	Providing an evaluation framework for mobile contact tracing solutions
Ferretti et al. [50]	Bluetooth	Neighborhood	Weeks	Exploring the feasibility of different contact tracing solutions
Carli et al. [51]	Bluetooth	Neighborhood	Weeks	Proposing a Bluetooth low energy- (BTE-) based contact tracing approach
Leith and Farrell [52]	Bluetooth	Neighborhood	Weeks	Reporting the measurements of Bluetooth low energy (LE) in different environments
Brack et al. [53]	Bluetooth	Neighborhood	Weeks	Presenting a decentralized peer-to-peer contact tracing system
Bian et al. [54]	Other	Neighborhood	Weeks	Monitoring social distance at real time by magnetic-based system
Xiao et al. [55]	GPS/CDRs	Neighborhood	Weeks	Predicting risk of the community before the spread of COVID-19 from epicenter
Park et al. [56]	GPS/CDRs	Neighborhood	Weeks	Understanding privacy issues in disclosing the personal information of the infected
Berke et al. [57]	GPS/CDRs	Neighborhood	Weeks	Providing a secure approach to evaluate the risk of exposure to infected cases
Ye et al. [58]	GPS/CDRs	Community	Weeks	Estimating provided risk indices associated with a community based on mobile data
Kielienyu et al. [59]	GPS/CDRs	Community	Weeks	Predicting COVID-19 risk scores by model based on mobile crowd-sourced data

*(2) Outcomes*. The spatial coverage of two contact tracing ways (i.e., proximity- vs. location-based systems) may vary, while proximity-based solution straightforwardly records the in-person contacts between users [50,52] and location-based tracing estimates exposure risk to COVID-19 through calculating the colocation estimations between mobile users, i.e., two mobile users appear in the same location at the same time or within a short period [58,59]. Thus, proximity-based contact records are fine grained but sparse, as only a few people will always have Bluetooth on [60] and the sensing functioning of Bluetooth is capable within meters to match the ability of COVID-19 to spread person to person, but limited once one person does not turn on the Bluetooth. On the other hand, the location-based contact records are usually coarse grained in spatial and depending on the spatiotemporal granularity to define “locations” or a “colocation” event. To be detailed, when people are in close proximity, their devices communicate through exchanging encrypted tokens. Later, when one is tested positive, she can opt-in share the key of her anonymous token to the public, where the ones holding the corresponding token could decode it and get notified as a contact [50,52], while the location-based methods estimate the colocation exposure risk in a neighborhood/community space by collectively analyzing the interactions between people’s historical trajectories. For example, Xiao et al. [55] designed an AI predictive framework that screens human mobility at the urban neighborhood level and predicts infection risks. Berke et al. [57] proposed to divide the city into grids to assess and communicate users’ exposure risk by tracing intersections of the GPS trajectory with the infected on each spatial grid. Furthermore, the effectiveness evaluation of contact tracing is not always trustworthy [13]. The abovementioned works are either based on well-designed experiments under laboratory conditions [52] or run simulations with mobile data under assumptions [50], which were not verified in large-scale scenarios.

*(3) Limitations*. For contact tracing, the privacy issue is the major concern impeding the public’s willingness to participate in, despite that several privacy-preserving methods for data collection exist. Technically, few privacy-preserving standards have been pervasively adopted and the magnitude of the risk of indiscriminate data collection and chronic privacy breaches depends on the capabilities and attitudes of data managers, not users [61]. The privacy issue may also be compounded in communities with a high prevalence of mistrust of the government or healthcare institutions [62]. As a result, these communities may be less willing to share data and, at the same time, to pursue preventative strategies such as masking, distancing, and vaccination, placing them at higher risk for COVID-19 infection and more severe illness.

#### 3.4.4. Epidemiological Study

*(1) Design*. Epidemiological study collects the GPS locations or CDR data from massive users. However, the population scales under coverage of mobile sensing in these studies range from (*1) neighborhoods/cities* (N=14 of 27) [63– 76] to (2) *states/nation* (N=9 of 27) [77– 85] to (3) *multinations/global* (N=4 of 27) [86– 89] (see Table 7). The global human mobility surges the worldwide spread of the virus from one country to the others, while the variants of COVID-19 were naturally caused by the mutations within large infected populations. During the pandemic and the pervasive adoption of mobile devices, Internet tycoons have enabled large-scale mobility tracking and provided the aggregated mobility data usable. Some academic research obtains nonpublic data through collaboration with the industry and government; others have processed coarse grained (e.g., city-level statistic results) data available to the public. Platforms such as Google Mobility Report Google Mobility Report, https:// http://www.google.com/covid19/mobility/, Baidu Qianxi,https://qianxi.baidu.com/, Apple Mobility Trend Reports Mobility Trend Report,https://covid19.apple.com/mobility, and CDC COVID Data Tracker COVID Data Tracker,https://covid.cdc.gov/covid-data-tracker/, provide these large-scale mobility data, including aggregated counts of inflows and outflows between spatial regions over time series; some also provide the population activity index (e.g., traffic index) within the spatial areas (e.g., a city). Mobile-enabled epidemiological studies generally investigate the associations between mobile-measured human mobility and pandemic spread, sometimes combining with other external data (e.g., policy, social media, and vaccination data) [1,8].

**Table 7:** Study characteristics of epidemiological study literature.

Author	Data type	Population scale	Sensing duration	Description
Hao et al. [63]	GPS/CDRs	City	Weeks	Assessing intracity mobility for understanding virus spread using LBS data
Li et al. [64]	GPS/CDRs	Community	Months	Assessing the association between community mobility using Google Mobility Index
Kephart et al. [65]	GPS/CDRs	City	Months	Analyzing subcity population mobility and COVID incidence using LBS data
Martin et al. [66]	GPS/CDRs	City	Months	Evaluating social distancing strategies using detailed movement data in cities
Rader et al. [67]	GPS/CDRs	City	Months	Predict epidemiology with data on climate, population, mobility, and outbreak responses
Gondauri and Batiashvili [68]	GPS/CDRs	City	Months	Studying time-delayed impacts of pedestrians, traffic, and transit traffic on virus spreading
Cintia et al. [69]	GPS/CDRs	City	Months	Discovering the relationship between mobility flows and net reproduction using LBS data
Zhou et al. [70]	GPS/CDRs	City	Months	Building a transmission model for COVID-19 using mobile sighting data
Showalter et al. [71]	GPS/CDRs	City	Months	Understanding human mobility differences between tribal/nontribal and rural/urban
Gao et al. [72]	GPS/CDRs	County	Months	Understanding the mobility pattern changes with mobile data at county level
Xiong et al. [73]	GPS/CDRs	County	Months	Computing the origin-destination travel and infections using LBS data
Kishore et al. [74]	GPS/CDRs	County	Months	Capturing the contact patterns of COVID-19 transmission from aggregated LBS data
Pan et al. [75]	GPS/CDRs	County	Months	Constructing social distancing index by using LBS data to study location mobility
Sehra et al. [76]	GPS/CDRs	County	Months	Understanding the differences in human activity between workplace and residence
Vinceti et al. [77]	GPS/CDRs	State	Months	Relating mobile phone data to measure mobility restriction with the number of cases
Gao et al. [78]	GPS/CDRs	State	Months	Human mobility patterns changed during stay-at-home orders and reduced the cases
Unwin et al. [79]	GPS/CDRs	State	Months	Using mobility changes to capture the impact of NPIs on the transmission of COVID
Kraemer et al. [80]	GPS/CDRs	National	Months	Quantifying control measures and their impact on human mobility from open data
Tian et al. [81]	GPS/CDRs	National	Months	Investigating COVID-19 spread with human movement and public intervention data
Pepe et al. [82]	GPS/CDRs	National	Months	Aggregating mobility to monitor lockdown’s impact on the epidemic trajectory
Garcia-Cremades et al. [83]	GPS/CDRs	National	Months	Creating the decision support system for early prediction of the COVID-19 evolution
Kang et al. [84]	GPS/CDRs	National	Months	Monitor epidemic spreading using mobile phone visit data from SafeGraph
Chang et al. [85]	GPS/CDRs	National	Months	Linking the associations between mobility complexity and infection risks
Sirkeci and Yucesahin [86]	GPS/CDRs	International	Months	Predicting the COVID-19 spread based on migrant stock and travel data
Chatterjee et al. [87]	GPS/CDRs	International	Months	Using LSTM models to forecast the new cases and caused death
Chinazzi et al. [88]	GPS/CDRs	International	Months	Using the disease transmission model to project the impact of travel limitations
Liu et al. [89]	GPS/CDRs	International	Months	Leveraging mobility data from Baidu and Google to analyze flow intensities

*(2) Outcomes*. Population health outcomes of epidemiological study include the public health policy making and nonpharmaceutical interventions (NPIs) at various scales. For epidemiological study at the city scale [66,85], the main outcomes lie in assessing the efficiency of regional policies (e.g., social distancing) and identifying high-risk regions. For example, in the Boston metropolitan area, Martin-Calvo et al. [66] built colocation networks at three layers (i.e., community, households, and schools) to test the social distancing policy; results showed that most of the infections occur at community and households layers, where school closures might be ineffective and costly for the overall well-being. Chang et al. [85] proposed to figure out the inequities and gaps between races and socioeconomic groups with a mobility network model.

On state, nation, and global levels, mobile data has been collected to monitor large-scale human mobility to evaluate higher-level policies such as lockdown and travel bans. For example, using population migration data among cities collected by Baidu company, Kraemer et al. [80] verified that the spatial distribution of COVID-19 cases in China at both city and province levels is significantly correlated to human mobility. After the implementation of control measures, such as travel ban, such correlation dropped, which indicated that the drastic control measures have paid off. Chinazzi et al. [88] leveraged Baidu data plus global travel data to map other counties’ relative risk of case importation and simulated the travel and transmissibility reductions under international travel restrictions. Similarly, using CDRs in three regions of Italy, the timing and efficacy of the lockdown have been estimated to guide further restriction adjustment [77]. Pan et al. [75] constructed a social distancing index to quantify and understand the influence of policies on people’s dynamic social behaviors prolonged for 4 months.

*(3) Limitations*. The use of mobile data to inform COVID-19 epidemiological studies is after all a secondary use, compared to its primary use for location-based services. Up to now, few standardized frameworks have been protecting users’ privacy and confidentiality of such practices [8,61]. Not to mention that even anonymized and aggregated data can be reidentified to recover individuals’ trajectories under some circumstances [90]. There still needs actions to make mobile users decide how, when, and for what purposes the data could be collected or released, though the data is only used for research purposes, released to the public after location-based aggregation, and only statistical results are accessible [91].

Along with privacy, tracking COVID-19 trends on a global scale may underestimate prevalence in low- or middle-income countries, where testing is less widely available than in countries with more resources [92]. This can lead to unequal representation of populations in the data. If the data is then used to inform policy decisions, the severity of the pandemic may not be adequately addressed for populations who lack resources for mobile tracking, in addition to lacking resources for accurate case reporting [93].

## 4. Discussion

In this section, with identified limitations shown in Table 8, we highlight recent discoveries and viewpoints on implications, benefits, and limitations of mobile sensing practices.

**Table 8:** Summary on the limitations of each cluster of mobile sensing work.

	Imperfect data	System scalability	Privacy concern	Social equality
Remote detection		√	√	√
Long-term tracking	√	√	√	
Contact tracing	√	√	√	√
Epidemiological study	√		√	√

### 4.1. Technical Challenges and Opportunities

Technical limitations hindering the feasibility of using mobile sensing for COVID-19 include data quality and system adoption issues. For data quality, imperfect data is an inherent issue for mobile sensing, as it collects individuals’ data following a consent-based, opt-in standard from a wide variety of mobile devices. Such practice naturally leads to the data sparse, out-of-sync, or even missing on time scales and biased, heterogeneous over populations. We suggest that advances in data analytics and machine learning methods capable of handling sparse, heterogeneous, and multimodal mobile sensing data streams could be helpful. For system adoption, in this study, we can observe significant clustering phenomena of mobile sensing applications on time and population scales (see Figure 2). This phenomenon indicates that there is a tradeoff between the data granularity and population coverage of the currently used mobile devices, depending on the technological literacy and health literacy of the users. Arguably, mobile sensing at even larger scales (particularly in clinical settings) could be carried out on top of the next generation of sensors and sensing platforms. 

### 4.2. Clinical and Societal Implications

The use of mobile sensing to identify and track individuals with COVID-19 infection or at increased risk of infection has implications for clinicians. Patients can be monitored for ongoing symptoms or worsening the clinical status remotely, for example, after being discharged from a hospitalization for COVID-19. Remote monitoring can also help keep patients out of the hospital, reducing demand on the limited space and staff in the emergency department and hospital wards. However, data generated by mobile sensing would have to be timely and actionable, in order to be useful to clinicians, who are already overburdened in pandemic conditions. Public health professionals can also benefit from tracking of individuals and communities within the populations that they serve, but with similar attention to generating actionable data.

Implications of mobile sensing for healthcare systems include the potential to monitor and predict the spread of pandemics. Taking care of patients with COVID-19 infection and its complications requires significant investment of staff, supplies, space, and other limited resources. Mobile sensing that can aid predictions of impending surges in pandemic cases could provide healthcare systems and public health departments with an early warning signal to inform the allocation of resources, which can be challenging to mobilize.

From a health policy standpoint, efforts are needed to mitigate potential threats to privacy while also harnessing the benefits of mobile sensing technology. Pandemic control strategies at the local and national scale, such as closure of schools and businesses, have far-reaching social and economic consequences. Such decisions should rely on accurate epidemiologic data and prediction models. Attention to equity and health disparities is crucial, so that communities most at risk from pandemics are not further disadvantaged by unequal access to technology or excluded from algorithms used to inform resource allocation. Policy makers would need to consider strategies to promote technological literacy and health literacy in all communities, in particular those at high risk of harm from pandemics. In addition, involvement of community stakeholders in decision-making that balances risks and benefits is needed to build trust. Lessons learned from the current COVID-19 pandemic can inform planning for future pandemics, which will continue to be a concern as emerging infectious diseases arise.

## 5. Conclusions

Mobile sensing has shown its power to pervasively and effectively monitor COVID-19 in varying population scales and time duration. Existing works have demonstrated the potential of mobile sensing techniques to identify individuals’ infectious status through acoustic/wearable sensing and symptom self-reporting, to track the long-term self-reported symptoms and physiological data to monitor the progression or recovery from the disease, to estimate the exposure risk to COVID-19 and trace the spread among neighborhoods through mining colocation events from mobility traces, and to surveil the pandemic in city, state, nations, and global scales for public health policy making. For future research, we wish to see more works where computer scientists, clinicians, and epidemiologists design and implement the study collaboratively with experts in social science, public policy, and human factors to enable more effective, scalable, and socially equal mobile-based sensing systems for future needs.
